# Pilot Study of Mindfulness Training on the Self-Awareness of Motor Symptoms in Parkinson’s Disease – A Randomized Controlled Trial

**DOI:** 10.3389/fpsyg.2021.763350

**Published:** 2021-11-30

**Authors:** Timo Marcel Buchwitz, Franziska Maier, Andrea Greuel, Franziska Thieken, Kenan Steidel, Viktoria Jakobs, Carsten Eggers

**Affiliations:** ^1^Department of Neurology, University Hospital Marburg, Marburg, Germany; ^2^Department of Psychiatry, Medical Faculty, University Hospital Cologne, Cologne, Germany; ^3^Center for Mind, Brain and Behavior (CMBB), Universities Marburg and Gießen, Marburg, Germany

**Keywords:** self-awareness, anosognosia, Parkinson’s disease, mindfulness, randomized controlled trial

## Abstract

**Objective:** This study aims to evaluate feasibility and effects of a newly developed mindfulness intervention tailored to specific needs of patients with Parkinson’s disease (PD).

**Background:** The phenomenon of impaired self-awareness of motor symptoms (ISAm) in PD might be reduced by increasing patients’ mindfulness. A PD-specific mindfulness intervention has been developed and evaluated as a potential treatment option: IPSUM (“Insight into Parkinson’s Disease Symptoms by using Mindfulness”).

**Methods:** IPSUM’s effectiveness is evaluated by comparing an intervention with a waitlist-control group. Applying a pre-post design, patients were assessed before, directly after and 8weeks after treatment. The primary outcome was the change in a quantitative ISAm score from baseline to post-assessment. Secondary outcome measures were PD-related affective changes and neuropsychological test performance. Feasibility was evaluated *via* feedback forms.

**Results:** In total, 30 non-depressed and non-demented PD patients were included (intervention: *n*=14, waitlist-control: *n*=16). ISAm score did not change significantly, but the training group showed greater performance in sustained attention and language tasks over time. Additional changes included greater mindfulness as well as less sleeping problems and anxiety. Cognitive disturbances, apathy, and sleeping problems worsened only in the waitlist-control group. Patients’ feedback regarding the training concept and material was excellent.

**Conclusion:** Insight into Parkinson’s Disease Symptoms by using Mindfulness has not been capable of reducing ISAm in PD patients but appears to be a feasible and effective concept to, among others, support mental health in the mid-term. It has to be noted though that the study was stopped beforehand because of the SARS CoV-2 pandemic. The lack of findings might therefore be caused by a lack of statistical power. The need for further research to better understand the mechanisms of ISAm and its connection to mindfulness in PD is highlighted.

## Introduction

Over the last few years, research increasingly focused on the impairment of self-awareness in neurodegenerative disorders. Impaired self-awareness (ISA) is defined as a partial inability to perceive one’s neurological and neuropsychological symptoms (Prigatano et al., 2014) and has been associated with lower therapy adherence, higher patient mortality, and higher caregiver burden (Prigatano, 1999; Koltai et al., 2001; Appelros et al., 2007; Turró-Garriga et al., 2013). The phenomenon has already been described with respect to memory deficits in patients with Alzheimer’s disease (e.g., Shany-Ur et al., 2014). Besides awareness of cognitive deficits, in Parkinson’s disease (PD), research has focused on impaired self-awareness of motor symptoms (ISAm). ISAm is present if any PD motor symptom (hypokinetic and dyskinetic symptoms alike) is not perceived by a patient but is perceived by another more objective person like a caregiver or doctor. Therefore, ISAm exists if a patient states no functional impairment when in fact they are impaired, meaning they underestimate their level of functional impairment. As ISAm is a subjective experience, it can only be measured indirectly and is described along a continuum ranging from mild to moderate to severe forms (Maier et al., 2015; Maier and Prigatano, 2017). PD is classified as a movement disorder, but patients regularly also suffer from various nonmotor symptoms including sleep disturbances or hyposmia as well as psychiatric symptoms, e.g., depression, anxiety, and impulsivity (Postuma et al., 2015). Cognitive symptoms in PD are heterogeneous and comprise impairments of attention, executive functioning, memory, and visuospatial abilities. Only language abilities (except for word retrieval) are mostly unimpaired (Kalbe and Petrelli, 2014).

Impaired self-awareness of motor symptoms has been studied extensively in regards to levodopa-induced dyskinesias (ISAm-LID). It was found in up to 91% of patients with dyskinesias meaning that 91% of the sample did not perceive a dyskinetic movement (Vitale et al., 2001; Maier et al., 2015). It has been associated with higher disease duration, higher levodopa equivalent daily dose (LEDD), and predominantly left-sided symptoms (Amanzio et al., 2010; Pietracupa et al., 2013; Maier et al., 2016). In contrast to ISAm-LID, an impaired self-awareness for hypokinetic movements (ISAm-Hypo) was only found in up to 54% of patients in the medication ON-state and up to 55% in the medication OFF-state (Maier et al., 2015, 2016). In the OFF-state, ISAm-Hypo has been associated with left-sided disease onset and worse left-sided symptoms. These findings were not obtained in the medication ON-state (Maier et al., 2016). In previous studies, unnoticed symptoms were most commonly of mild severity. Pennington et al. (2020) hypothesize that early symptoms are often underreported due to their “insidious onset and lack of impact on overall motor functioning” (p. 6), while the lack of awareness of more severe symptoms in later disease stages might be caused by impairments of neural processes.

The discrimination between ISAm-LID and ISAm-Hypo is assumed to be based on different pathogenic mechanisms and networks (see Pennington et al., 2020 for a detailed overview). In general, areas of the prefrontal cortex have been linked to ISAm. Specifically, hypometabolism in the right inferior frontal gyrus and the right insula have been linked to ISAm-Hypo in PD (Fotopoulou et al., 2010; Moro et al., 2016).

Originally, the concept of mindfulness is rooted in Buddhism. In order to further study its usefulness on a scientific level, many different definitions were created. One that has been widely used was created by Kabat-Zinn (2004, S. 4) who described mindfulness as a specific way of paying attention: “on purpose, in the present moment, and nonjudgmentally.” It may be enhanced by regular meditation and yoga practice or by maintaining a mindful attitude during daily life activities (Kabat-Zinn, 2013; Schug, 2016). Keng et al. (2011) summarized that mindfulness is positively associated with a range of variables indicating wellbeing and mental health. It is also negatively associated with psychiatric symptoms indicating mental illness (e.g., depression or social anxiety). Therefore, mindfulness appears to be linked, among other things, to higher quality of life, autonomy, and vitality. Mindfulness meditation might also enhance cognitive performance of attention, self-reflection, working memory, or even executive functioning (Chiesa et al., 2011; Sedlmeier et al., 2012). Additionally, especially long-term meditators showed an improved awareness and interpretation of body states (Tang et al., 2015), higher sensitivity for body sensations and higher coherence of emotional perception and physiological arousal (Sze et al., 2010).

This is underlined through several yoga studies which also reported positive implications regarding body awareness, self-representation, and acceptance toward oneself (Emavardhana and Tori, 1997; Daubenmier, 2005; Impett et al., 2006; Dittmann and Freedman, 2009). Behavioral findings are supported by various neurobiological studies. For example, Fox et al. (2014) conducted a meta-analysis including 21 studies, which identified differences in volume and density of gray and white matter in various regions, including the bilateral insula, somatomotoric cortices, and the anterior and mid-cingulate gyrus. The identified regions have also been linked to abilities of introspection as well as metacognitive and body-oriented perception. Although most previous studies have focused on long-term meditators, changes might already be significant after approximately 8weeks of mindfulness training (Gotink et al., 2016).

A variety of mindfulness-based interventions have been previously developed and applied in a clinical context. In research, clinical studies applying a pre-post design to examine the effects of these interventions in the PD population have been conducted before. Some of them used an adaptation of the mindfulness-based stress reduction program (MBSR; Pickut et al., 2015; Cash et al., 2016; Dissanayaka et al., 2016), while others focused on elements of conventional mindfulness training (e.g., yoga; Advocat et al., 2016; Kwok et al., 2019). Training duration usually lasted between 6 and 10weeks. In most studies, an increase of mindfulness and quality of life and a reduction of negative emotional states like depression, anxiety, and stress were reported (Pickut et al., 2015; Advocat et al., 2016; Cash et al., 2016; Dissanayaka et al., 2016; Rodgers et al., 2019). Interestingly, some studies reported a reduction in motor impairment level in PD (Pickut et al., 2015; Dissanayaka et al., 2016), especially after patients’ participation in a yoga-based intervention (Kwok et al., 2019). It has to be noted that the quality of many mindfulness intervention studies in PD has been criticized before (McLean et al., 2017). Despite methodological flaws of existing studies, mindfulness is still expected to be beneficial for PD patients, e.g., in terms of stress reduction (van der Heide et al., 2020).

To the best of our knowledge, no cross-sectional nor longitudinal study is available regarding the possible connection between mindfulness and ISA in PD. ISA has been associated with lower therapy adherence, higher patient mortality, and higher caregiver burden in other diseases. Assuming these consequences are relevant for PD patients as well, mindfulness might be helpful to improve ISAm and positively influence the course of the disease. In addition, it remains unclear whether the training of mindfulness might also improve cognitive functioning in PD as no study included extensive, objective neuropsychological testing.

The main aim of this study was to examine possible effects of mindfulness training on ISAm in PD. The aforementioned consequences of ISA (Prigatano, 1999; Koltai et al., 2001; Appelros et al., 2007; Turró-Garriga et al., 2013) might also be applicable in terms of ISAm even though no study did systematically examine their generalizability. We assume that therapy adherence might be lower in regard to medication as patients might not see the need to adhere to their treatment plans if they are convinced that the impairment is non-existent. Overestimation of one’s own capabilities might also lead to more injuries and as a consequence to higher mortality and caregiver burden. Lastly, the term of caregiver burden comprises interpersonal conflicts between the caregiver and the patient caused by different evaluations of impairment.

Additionally, the effectiveness of mindfulness regarding the improvement of cognitive abilities and changes of PD-related affective variables was studied. We developed a new mindfulness-based intervention called IPSUM (an acronym for “Insight into Parkinson’s Disease Symptoms by using Mindfulness”). The 8-week long intervention is influenced by existing mindfulness programs, which have been previously applied in PD research and respective good practices to facilitate mindfulness training for PD patients. Thus, IPSUM has been developed with consideration of PD-specific challenges, such as reduced attention span, impaired executive functioning, and lesser mobility. The program aims to increase patients’ mindfulness, quality of life, and their ability of self-awareness. Based on the described literature, we hypothesize a positive change in self-awareness by increasing patients’ awareness of the present moment in general, as well as their acceptance toward individual perceptions of body movements and posture. These processes might be facilitated by regular attention training *via* mindfulness meditation and improvement of emotion regulation abilities. In addition, we hypothesize a general reduction of negative affective states (like depression, anxiety, and apathy) and self-reported cognitive impairment (cognitive failures and dysexecutive impairment) as well as general improvements of objective cognitive performance.

## Materials and Methods

This study was a mixed-method, randomized clinical trial comparing an intervention and a waitlist-control group in a pre-post design. The current paper presents the study’s quantitative data and findings. Neurobiological and qualitative data will be analyzed and reported separately. The intervention consisted of a newly developed 8-week mindfulness training program, which was adapted to specifically address the needs of patients with Parkinson’s disease. The study was conducted over a 12-month period in 2019. Due to the SARS CoV-2 pandemic and associated nationwide regulations, data collection was ended earlier than previously planned. The study has been approved by the local ethics committee of the University Hospital Marburg (study number: 119/18) and registered at the German Clinical Trials Register (DRKS00015807). A detailed description of the methodology has been previously published (Buchwitz et al., 2020).

### Participants

Patients with idiopathic PD [diagnosed according to the Movement Disorder Society criteria (Postuma et al., 2015)] between 45 and 85years of age were recruited from the Department of Neurology, University Hospital Marburg, Germany, and regional patient support groups. Patients’ eligibility to undergo a magnetic resonance imaging (MRI) scan was also checked. However, this was not an exclusion criterion. All patients had to give written informed consent prior to participation in accordance with the Declaration of Helsinki.

Exclusion criteria were as:

- Moderate depression measured by the Beck Depression Inventory-2 (BDI-II) and defined as a score >19 (Beck et al., 1996; Hautzinger et al., 2006),
- Dementia measured by the Parkinson Neuropsychometric Dementia Assessment (PANDA) and defined as a score <15 (Kalbe et al., 2008),
- Clinical diagnosis of additional severe neurological or psychiatric disorders,
- Advanced disease defined as stage 5 on the Hoehn and Yahr (1967) scale due to an estimated incapability to perform the intervention exercises,
- Prior regular experience in meditation or yoga as this study seeks to examine mindfulness novices, and
- Changes of antiparkinsonian medication within 2weeks prior to baseline measurement.

### Study Design

**Figure 1:**
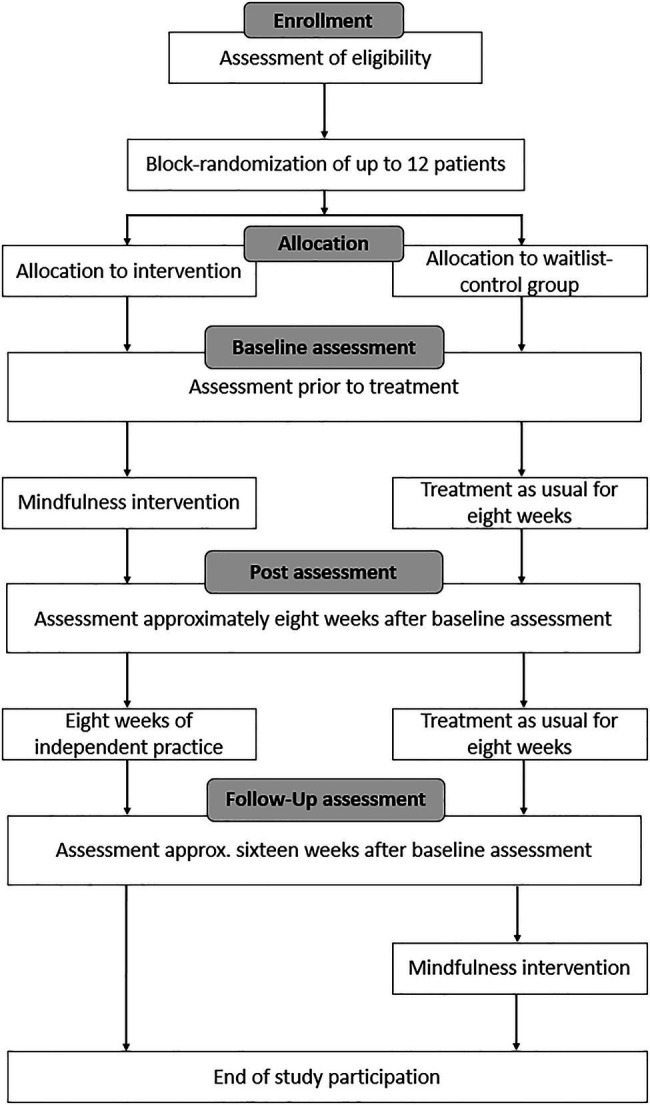
Course of the study. This figure has been adapted from a previous publication by Buchwitz et al. (2020).

Group randomization was performed several times over the course of the study while the recruitment process was ongoing. After about 10 patients gave written informed consent, available patients were randomized to either the intervention group or the waitlist-control group using Microsoft Excel. The randomized patients started to complete the intervention protocol shortly after to avoid the risk of study drop-outs due to long waiting times. In total, randomization was performed four times, resulting in four intervention and waitlist-control group pairs. All patients were measured three times: at baseline, after 8weeks (post-measurement) and again after an additional 8weeks (follow-up measurement). The intervention took place over the course of 8weeks between the first two measurements. Patients of the control group did not take part in the intervention and were treated as usual during this time, meaning that they were asked to carry-out their everyday routine unchanged. The study team did not contact the patients over the course of their study participation except for making appointments for the three measurements. The control group patients were offered to participate in the intervention after the study had ended without additional measurements. Figure 1 gives a comprehensive overview of the course of the study.

During in-person meetings at all three measuring points, ISAm and disease severity were evaluated. Cognitive functioning was assessed using an elaborate neuropsychological test battery to allow for classification of mild cognitive impairment (MCI). Data were collected by a trained medical student (V.J.), who was blinded for the patients’ group assignment. In advance to these meetings, a set of questionnaires was sent to the patients *via* mail to enable a completion at home and to reduce the duration of the recurring meetings. Independent of their study group, all patients completed the measurements at all three measuring points in the same order. All measurements were performed in the medication ON-state only, as measurements in the OFF-state would have been too much of a strain for most patients.

A recently published review reports scarce information regarding the assessment of ISAm in longitudinal study designs (Pennington et al., 2020). The results of a power analysis based on the results reported by Maier et al. (2015) suggested that a total of up to 166 patients (83 patients per group) might be needed to detect a significant effect of the primary outcome. Because of the ongoing SARS CoV-2 pandemic, the study was prematurely terminated.

### Outcome Measures

In congruence with the aim of this study, the primary outcome was the change of a global ISAm score from baseline to post-measurement. Secondary outcomes included neuropsychological test performance, changes of several PD-related symptoms, and affective states. All outcomes were tested for within-group changes after examination of potential baseline differences.

#### Primary Outcome

After watching video clips demonstrating the correct execution of each task of the ISAm measurement recently developed and validated by Maier et al. (2015), the patient was asked to repeat the tasks him/herself. The following 15 motor symptoms were examined within five tasks:

- Sitting on a chair: resting tremor (left and right hand), dyskinesia
- Right hand pronation-supination: speed and amplitude of movement, dyskinesia
- Left hand pronation-supination: speed and amplitude of movement, dyskinesia
- Arising from a chair: resting tremor (left and right hand), dyskinesia
- Walking down an aisle: resting tremor (left and right hand), dyskinesia

The motor symptoms can be distributed across four subscales: bradykinesia, dyskinesia, resting tremor right hand, and left hand. The patients rated their performance on a dichotomous scale concerning presence of deficit and each patient answered 15 questions with either yes or no. The whole procedure was videotaped and later on, motor symptoms were evaluated by two independent raters. ISA is noted each time a discrepancy occurs. A relevant discrepancy occurred if the patient did not see any impairment, although the raters did. By merging the 15 motor symptoms assessed within the five tasks, the total score of ISAm is calculated. In the medication ON-state, the ISAm total score can be divided into subscores for impaired self-awareness of hypokinetic symptoms (ISAm-Hypo for tremor and bradykinesia) and dyskinetic symptoms (ISAm-LID). For each motor task, the independent raters checked for a presence of motor symptoms in general on the same dichotomous scale. In case of impaired movements, they defined the severity of motor impairment according to Unified Parkinson’s Disease Rating Scale part III (UPDRS-III; values range 0–4; 0=normal/absent; 1=mild; 2=moderate; 3=severe; and 4=unable to perform). These are accumulated to a total score. This method allowed to compare the severity of motor impairment as measured by the ISAm tool to the severity of ISAm. Both scores can vary between 0 and 60. It has to be noted that the motor impairment as measured by the ISAm tool comprises fewer motor symptoms than the UPDRS-III. In the following chapters, we therefore discriminate between general motor impairment and ISAm motor impairment. Average scores of both raters are calculated for further statistical analyses.

#### Secondary Outcomes

At the baseline visit, each patient’s cognitive performance was intensively evaluated. For this purpose, we compiled a test battery to apply a level II-assessment of MCI by taking the guidelines of diagnostic criteria for MCI in PD (Litvan et al., 2012) into account. For each cognitive domain, two different neuropsychological tests were used. Table 1 specifies the applied tests. MCI was diagnosed if baseline performance was at least 1.5 standard deviations below the mean score of a test specific norm sample. At post- and follow-up measurement, a shortened version of the neuropsychological test battery was applied to minimize patient strain. Tests listed in the column “Test I” were used repeatedly and serve as secondary outcomes. In addition to the test battery, each patient completed the Montreal Cognitive Assessment [MoCA (Nasreddine et al., 2005)] at each measuring point. For each assessment, another parallel version of the MoCA was applied.

**Table 1:** Overview of applied neuropsychological tests.

Domain	Tests applied repeatedly	Tests only applied at baseline
Attention/Working memory	TAP: Sustained attention^*^	WMS-R: Digit span forwards/backwards^*^
Executive Functioning	RWT: Lexical verbal fluency	Trail Making Test A+B^*^
	RWT: Alternating lexical verbal fluency RWT: Semantic verbal fluency	
	RWT: Alternating semantic verbal fluency^*^	
Language	WAIS-IV: Vocabulary^*^	WAIS-IV: Similarities^*^
Memory	VLMT: Learning performance	ECFT-MI: Recognition Trial^*^
	VLMT: Delayed recall^*^	
	VLMT: Recognition Trial	
Visuospatial Ability	WMS-R: Spatial Span forwards/backwards^*^	ECFT-MI: Matching Trial^*^

ECFT-MI, Extended Complex Figure Test – Motor Independent Version; RWT, Regensburg verbal fluency test; TAP, test battery for attention; VLMT, Verbal learning and memory test; WAIS-IV, Wechsler Adult Intelligence Scale – Fourth Edition; and WMS-R, Wechsler Memory Scale-Revised. This table has been adapted from a previous publication by Buchwitz et al. (2020).

* Tests used to check for mild cognitive impairment.

Patients repeatedly completed a set of questionnaires at home to assess changes of PD-related symptoms and affective states. Symptoms of interest, the applied questionnaires, and their respective subscales, score range, and score interpretations are described in Table 2.

**Table 2:** Overview of questionnaire subscales, score range, and score interpretation.

Construct	Questionnaire	Subscales	Score range	Higher score interpretation
Apathy	AES (Marin et al., 1991)	None	18–72	Higher apathy
Depression	BDI-2	None	0–63	Higher depression
Cognitive Failures	CFQ (Broadbent et al., 1982)	Forgetfulness, Distractibility, Trigger	0–100	Higher impairment
Dysexecutive Functioning	DEX (Wilson et al., 1998)	Inhibition, Targeted action, Social regulation, Abstract thinking	0–80	Higher impairment
Mindfulness	FFMQ-D (Michalak et al., 2016)	Observing, Describing, Acting with Awareness, Non-judging of inner experience, Non-reactivity to inner experience	0–195	Higher mindfulness
Quality of life	PDQ-39 (Berger et al., 1999)	Mobility, Activities of daily living, Emotional wellbeing, Stigma, Social support, Cognition, Communication, Bodily discomfort	0–100	Lower quality of life
Sleep quality	PDSS-2 (Trenkwalder et al., 2011)	None	0–60	Worse sleep
Stress	PSQ-20 (Fliege et al., 2005)	Worries, Tension, Demands, Joy	0–100	Higher stress
Impulsivity	QUIP (Probst et al., 2014)	Gambling, Sex, Buying, Eating, Hobby, Punding, Medication	0–140	Higher impulsivity
Anxiety	STAI (Spielberger, 2010)	State, Trait	20–80	Higher anxiety

AES, Apathy Evaluation Scale; BDI-2, Beck Depression Inventory-2; CFQ, Cognitive Failures Questionnaire; DEX, Dysexecutive Questionnaire; FFMQ-D, Five Facet Mindfulness Questionnaire – German Edition; PDQ-39, Parkinson’s Disease Quality of Life Questionnaire-39; PDSS-2, Parkinson’s Disease Sleep Scale-2; PSQ-20, Perceived Stress Questionnaire-20; QUIP, Questionnaire for Impulsive-Compulsive Disorders in PD; and STAI, State-Trait Anxiety Inventory.

#### Feedback and Adherence Measures

For each patient of the intervention group, participation in the training sessions was recorded. To track the amount of mindfulness practice at home, patients were asked to complete homework exercises and to note all additional formal mindfulness exercise in a daily documentary sheet. Patients were asked to continue their daily documentation of mindful practice for eight additional weeks (until follow-up assessment). Based on this information, the amount of weekly formal mindfulness practice could be calculated.

To gain insight into the intervention’s feasibility, all patients who successfully finished the training program (including members of the former control group) were asked to complete an anonymous feedback questionnaire directly after the intervention had ended, which asked for their feedback regarding the quality and content of the training program, their training group, the instructor, their success with performing mindful exercises, and the quality of the audio-CDs for guided exercises at home. Ratings were measured using a 5-point Likert scale (ranging from −2: totally disagree to +2: totally agree). They were also asked for a total rating of the training program and the audio-CDs (ranging from 1: very good to 6: insufficient).

### Intervention Protocol

The intervention consisted of a newly developed mindfulness training, which has been designed to fit the specific needs of patients with Parkinson’s disease. Among others, adaptations comprised a shortened theoretical input to account for possible cognitive impairment. Yoga was performed on a chair because of potential mobility issues and gentle movement were included in the guided sitting meditation to prevent the patient from falling asleep.

The group-based training was designed for 4–6 patients, lasted 8weeks and consisted of eight weekly sessions of approximately 2h of duration. The intervention was instructed by a psychologist (main author T.M.B.). He designed the main part of the intervention concept and has extensive theoretical and academic knowledge of the literature regarding mindfulness and various established therapy programs like Acceptance and Commitment Therapy. He also had long-year practical experience with mindfulness and meditation techniques. To ensure adherence to the training protocol, power point presentations in combination with session-specific checklists were used. To facilitate mindfulness practice at home, the patients were asked to complete formal and informal mindfulness tasks as homework. Each patient received two audio-CDs with guided instructions as well as written summaries of all relevant information as additional support. For more detailed information about the intervention protocol, please refer to the previous publication (Buchwitz et al., 2020).

### Statistical Analysis

Data were analyzed using SPSS, version 27.0 (IBM Corp., Armonk, New York). Level of significance was set at 5% for all outcomes. For the total scores of ISAm and ISAm motor impairment, intraclass correlation coefficients [ICC; absolute agreement and average-measures] were calculated to determine the degree of agreement between independent raters. For both variables, average scores of both ratings were computed. Additionally, to include the degree of general ISAm motor impairment as an influential factor for potential ISAm score changes, ISAm percentage scores were calculated and analyzed. Therefore, total ISAm values were divided by total ISAm motor impairment values multiplied by 100. All variables were tested for normal distribution using Shapiro–Wilk test. Demographic and baseline characteristics were compared using Mann–Whitney *U* tests as a nonparametric alternative, because of the normal distribution assumption being violated for some characteristics. To analyze changes of the primary and secondary outcomes, the Friedman’s test for dependent measurements was used. Pairwise comparison *post-hoc* tests were performed using Dunn-Bonferroni tests. Due to the experimental character and the low sample size of this pilot study, no adjustment for multiple testing was applied.

Per-protocol analysis was used to evaluate the efficacy of mindfulness training on ISAm in a controlled sample of patients. However, the reason and time of drop out were documented, if a patient could not conclude his or her study participation. The method of last-observation-carried forward was applied to treat missing values.

## Results

**Figure 2:**
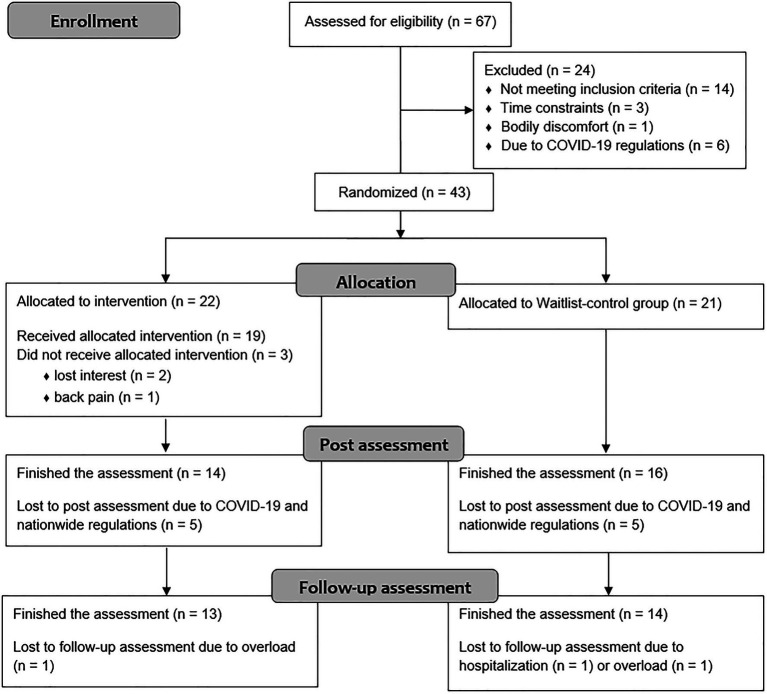
CONSORT diagram.

Initially, 67 patients were interested in study participation and tested for eligibility. Twenty-four patients did not meet inclusion criteria or declined participation before randomization due to various reasons. After randomization, three participants quit study participation. Ten patients were not able to complete post-measurement due to the SARS CoV-2 pandemic and associated nationwide regulations in Germany and were therefore excluded from the study. Figure 2 provides a more detailed CONSORT diagram.

In total, 30 patients (training group: *n*=14, waitlist group: *n*=16) completed post-measurement and were therefore included in the analyses. Twenty-seven patients (training group: *n*=13, waitlist group: *n*=14) participated in follow-up measurement and successfully completed the study protocol. Follow-up data for three patients were imputed. It also has to be noted that five missing questionnaire total score values (due to insufficient completion of questionnaires) have been imputed to maximize the number of data sets. Table 3 summarizes sample characteristics. At baseline, no significant differences were found between the intervention and waitlist-control group regarding neither demographic characteristics nor primary or secondary outcomes, indicating successful randomization of patients (see Appendix Table A). No adverse events occurred over the course of the study.

**Table 3:** Baseline sample characteristics for both groups and in total.

Baseline characteristics	Overall (*n*=30)	TRAINING (*n*=14)	WAITLIST (*n*=16)	*U*	*p*
Age in years, Median (range)	64.50 (46–80)	60.50 (46–79)	66.50 (54–80)	67.50	0.064
Hoehn & Yahr, Median (range)	2 (1–3)	2.00 (1–3)	2.00 (2–3)	99.00	0.608
UPDRS motor score, Median (range)	31.50 (7–49)	32.00 (7–48)	31.00 (21–49)	110.50	0.951
MoCA total score, Median (range)	27.00 (23–30)	25.00 (24–30)	27.50 (23–30)	88.00	0.334
LEDD score, Median (range)	626.0 (150–1,505)	487.5 (150–1,505)	827.5 (150–1,500)	74.50	0.120
Education in years, Median (range)	15.50 (10–21)	15.50 (10–21)	15.50 (10–20)	107.00	0.854
				***χ*** ^**2**^	***p***
Gender, Male/female, *n*	15/15	8/6	7/9	0.536	0.715
Deep brain stimulation, Yes/no, *n*	2/28	1/13	1/15	0.010	1.000
MCI, Yes/no, *n*	5/25	2/12	3/13	0.107	1.000

TRAINING, intervention group; WAITLIST, waitlist-control group; SD, Standard deviation; *n*, number of patients; UPDRS, Unified Parkinson’s Disease Rating Scale; MoCA, Montreal Cognitive Assessment; LEDD, Levodopa equivalent daily dose; and MCI, mild cognitive impairment.

### Medication, Motor Impairment, and Impaired Self-Awareness of Motor Symptoms

Levodopa equivalent daily dose increased over time in both the training group [*χ*^2^(2)=8.375, *p*=0.015] and the control group [*χ*^2^(2)=9.333, *p*=0.009]. Interestingly, *post-hoc* comparisons of time points did not reveal any significant change for either group. Motor impairment according to the UPDRS part III did not significantly change for any study group.

Interrater reliability at all three visits was high for total severity of ISAm (baseline ICC=0.833, post-ICC=0.894, and follow-up ICC=0.863) and good for ISAm motor impairment (baseline ICC=0.754, post-ICC=0.847, and follow-up ICC=0.778). During baseline and post-assessments, all patients showed at least one motor symptom. At follow-up, one patient of the training group did not show any motor impairment (as measured by the ISAm tool) during the assessment of ISAm. Additionally, at baseline, no patient of the control group showed left hand tremor. All other symptoms were present in at least one patient at all assessment points in time. In the training group, between 78.55 and 92.31% of patients showed ISAm at the different time points. In the control group, frequency ranged from 93.75 to 100%. Detailed results can be found in Table 4.

**Table 4:** Results of the ISAm testing.

	TRAINING (*N*=14)				WAITLIST (*N*=16)			
	Patients showing motor symptom	Patients not perceiving a symptom	Severity of motor symptoms shown	Unperceived motor symptoms	Patients showing motor symptom	Patients not perceiving a symptom	Severity of motor symptoms shown	Unperceived motor symptoms
	*N*	*N* (%)	*N*	*N* (%)	*N*	*N* (%)	*N*	*N* (%)
*Baseline*								
Total ISAm	14	11 (78.57)	65	36 (55.38)	16	16 (100)	85	50 (58.82)
Dyskinesia	7	6 (85.71)	17	13 (76.47)	10	9 (90.00)	22	14 (63.64)
Hypokinesia	14	9 (64.29)	47	22 (46.81)	16	14 (87.50)	58	34 (58.62)
Tremor right hand	2	2 (100)	4	2 (50.00)	2	2 (100)	4	3 (75.00)
Tremor left hand	1	0 (0)	1	0 (0)	0	0 (0)	0	0 (0)
Bradykinesia	14	8 (57.14)	38	19 (50.00)	16	13 (81.25)	54	31 (57.41)
*Post*								
Total ISAm	14	11 (78.57)	61	34 (55.74)	16	16 (100)	79	46 (58.23)
Dyskinesia	7	5 (71.43)	15	10 (66.67)	8	5 (62.50)	17	9 (52.94)
Hypokinesia	13	10 (76.92)	46	24 (52.17)	16	15 (93.75)	60	36 (60.00)
Tremor right hand	2	2 (100)	3	2 (66.67)	2	1 (50.00)	3	1 (33.33)
Tremor left hand	1	0 (0)	1	0 (0)	2	1 (50.00)	3	2 (66.67)
Bradykinesia	13	10 (76.92)	39	20 (51.28)	16	15 (93.75)	51	32 (62.75)
*Follow-Up*								
Total ISAm	13	12 (92.31)	50	34 (68.00)	16	15 (93.75)	72	50 (69.44)
Dyskinesia	8	7 (87.50)	11	11 (100)	9	9 (100)	18	17 (94.44)
Hypokinesia	13	11 (84.62)	39	25 (64.10)	16	16 (100)	54	37 (68.52)
Tremor right hand	1	0 (0)	1	0 (0)	1	1 (100)	1	1 (100)
Tremor left hand	1	0 (0)	1	0 (0)	1	1 (100)	1	1 (80.00)
Bradykinesia	13	11 (84.62)	34	22 (64.71)	16	14 (87.50)	48	29 (60.42)

TRAINING, intervention group; WAITLIST, waitlist-control group; and ISAm, Impaired self-awareness of motor symptoms in Parkinson’s disease.

Longitudinal analysis revealed no significant group differences at post- or follow-up assessment for ISAm total score or any ISAm subscale (see Table 5). However, ISAm motor impairment did change over the course of the study in both the control group [*χ*^2^(2)=11.288, *p*=0.004] and the training group [*χ*^2^(2)=8.346, *p*=0.015]. For the control group, significant differences were observed from baseline to post-assessment (*z*=0.938, *p*=0.008, *r*=0.23) as well as baseline to follow-up assessment (*z*=1.031, *p*=0.004, *r*=0.26). Changes within the training group were only significant from baseline to follow-up assessment (*z*=1.036, *p*=0.06, *r*=0.26). Total ISAm percentage scores did not change significantly in any study group.

**Table 5:** Absolute values for ISAm total and subscale scores, total ISAm percentage scores, levodopa equivalent daily dose, and motor symptom severity for all measurement points.

Variable name	Group	Baseline	Post	Follow-up	Within-group change	
		Median (range)	Median (range)	Median (range)	*χ* ^2^	*p*
ISAm total score	TRAINING	4.25 (0–11)	4.25 (0–11.50)	3.25 (0–10)	0.808	0.668
	WAITLIST	5.25 (1–11)	4.25 (1–10.50)	4.75 (1–10.50)	3.846	0.146
Impaired self-awareness (ISA) motor impairment score	TRAINING	8.50 (1–14.50)	7.25 (1.50–16.50)	5.00 (0–12.50)	8.346	0.015^*^
	WAITLIST	8.25 (5.50–14.50)	6.75 (3–13)	6.25 (4.50–12)	11.288	0.004^*^
Subscore Dyskinesia	TRAINING	0.50 (0–11)	0.25 (0–8)	0.75 (0–3)	1.220	0.543
	WAITLIST	1.00 (0–5.50)	0.00 (0–4)	0.75 (0–4.50)	5.905	0.052
Subscore Hypokinesia	TRAINING	2.50 (0–10.50)	2.50 (0–7)	2.50 (0–10)	0.542	0.763
	WAITLIST	4.25 (0–7.50)	3.25 (0–10.50)	3.25 (0–10.50)	0.400	0.819
Subscale Tremor right hand	TRAINING	0.00 (0–2.50)	0.00 (0–1.50)	0.00 (0–2)	0.800	0.670
	WAITLIST	0.00 (0–3)	0.00 (0–1)	0.00 (0–1.50)	0.737	0.692
Subscale Tremor left hand	TRAINING	0.00 (0–0.50)	0.00 (0–0.50)	0.00 (0–0.50)	1.000	0.607
	WAITLIST	0.00 (0–0.50)	0.00 (0–2)	0.00 (0–1)	5.200	0.074
Subscale Bradykinesia	TRAINING	2.00 (0–10.50)	2.25 (0–6)	2.50 (0–10)	0.776	0.679
	WAITLIST	3.75 (0–7.50)	2.75 (0–10.50)	3.00 (0–10.50)	0.327	0.849
LEDD	TRAINING	440 (150–1,505)	440 (150–1,505)	450 (150–1,505)	8.375	0.015^*^
	WAITLIST	827.50 (150–1,500)	827.50 (150–1,500)	827.50 (150–1,500)	9.333	0.009^*^
UPDRS motor impairment	TRAINING	32 (7–48)	35 (9–49)	32 (6–55)	1.469	0.480
	WAITLIST	31 (21–49)	32 (18–51)	33.50 (18–89)	0.102	0.950
Total ISAm percentage scores	TRAINING	54.89 (0–100)	61.90 (0–81.25)	73.26 (0–100)	1.472	0.479
	WAITLIST	63.33 (14.29–100)	59.82 (15.38–100)	65.15 (15.38–100)	2.847	0.241

TRAINING, intervention group; WAITLIST, waitlist-control group; ISAm, Impaired self-awareness of motor symptoms in Parkinson’s disease; UPDRS, Unified Parkinson’s Disease Rating Scale; LEDD, Levodopa equivalent daily dose; and SD, standard deviation.

* *p*<0.05.

### Neuropsychological Test Performance

General level of cognition measured by the MoCA did not change over time in any study group. For two of the more elaborate neuropsychological tests, significant differences in performance over the course of the study were observed in the training group but not the control group (see Table 6). For once, number of errors made in the sustained attention task of the test battery for attention (TAP) decreased [*χ*^2^(2)=7.860, *p*=0.02]. Changes from baseline to post-assessment (*z*=0.786, *p*=0.038, *r*=0.21) as well as changes from baseline to follow-up assessment (*z*=0.821, *p*=0.03, *r*=0.22) reached level of significance following a trend of reduction.

**Table 6:** Neuropsychological test performance for all measurement points.

Test	Subtest	Group	Baseline	Post	Follow-up	Within-group change	
			Median (range)	Median (range)	Median (range)	*χ* ^2^	*p*
RWT	Lexical	TRAINING	18.00 (7–27)	19.00 (6–35)	17.50 (8–33)	4.000	0.135
		WAITLIST	17.00 (4–31)	17.00 (10–25)	15.00 (8–27)	2.772	0.250
	Lexical alternating	TRAINING	21.00 (9–36)	21.50 (10–37)	21.50 (11–35)	1.480	0.477
		WAITLIST	20.50 (14–28)	21.50 (11–28)	22.00 (12–28)	0.453	0.797
	Semantic	TRAINING	30.50 (19–50)	34.50 (19–59)	33.50 (18–55)	3.880	0.144
		WAITLIST	30.50 (7–50)	32.00 (20–45)	32.50 (17–45)	1.300	0.522
	Semantic alternating	TRAINING	21.00 (12–34)	22.00 (16–37)	22.50 (12–33)	1.077	0.584
		WAITLIST	19.00 (12–27)	21.00 (15–25)	20.00 (15–26)	1.051	0.591
TAP	Total omissions	TRAINING	7.00 (0–22)	7.00 (0–22)	4.50 (0–21)	2.085	0.353
		WAITLIST	4.00 (0–21)	3.50 (0–33)	1.00 (0–20)	2.172	0.337
	Total errors	TRAINING	4.50 (0–19)	2.00 (0–8)	1.00 (0–9)	7.860	0.020^*^
		WAITLIST	2.00 (0–28)	2.00 (0–9)	1.00 (0–14)	3.304	0.192
VLMT	Learning performance	TRAINING	39.00 (26–61)	42.00 (24–62)	39.50 (24–65)	1.962	0.375
		WAITLIST	41.00 (29–55)	39.50 (25–61)	47.00 (26–61)	1.869	0.393
	Delayed recall	TRAINING	7.00 (0–15)	7.00 (1–15)	7.00 (2–15)	0.619	0.734
		WAITLIST	8.00 (1–12)	7.00 (3–15)	7.50 (2–15)	0.241	0.886
	Recognition	TRAINING	9.00 (−2–15)	8.50 (−11–15)	6.00 (−9–15)	3.040	0.219
		WAITLIST	11.00 (−5–15)	10.50 (2–15)	8.00 (−8–15)	4.456	0.108
WAIS	Vocabulary	TRAINING	45.00 (27–57)	51.00 (33–57)	50.00 (30–57)	8.769	0.012^*^
		WAITLIST	45.50 (14–53)	44.50 (19–55)	43.50 (26–55)	1.750	0.417
WMS	Spatial forward	TRAINING	8.00 (4–9)	7.00 (5–10)	7.00 (5–10)	0.341	0.843
		WAITLIST	7.00 (5–10)	7.00 (5–10)	8.00 (4–10)	2.000	0.368
	Spatial backward	TRAINING	6.00 (5–9)	6.50 (5–11)	7.00 (5–11)	2.711	0.258
		WAITLIST	6.50 (4–9)	6.50 (5–9)	6.00 (4–9)	0.553	0.758

TRAINING, intervention group; WAITLIST, waitlist-control group; SD, Standard deviation; RWT, Regensburg verbal Fluency test; TAP, test battery for attention; VLMT, Verbal learning and memory test; WAIS, Wechsler Adult Intelligence Scale – Fourth Edition; and WMS, Wechsler Memory Scale-Revised.

* *p*<0.05.

In the language subtest of the fourth edition of the Wechsler Adult Intelligence Scale (WAIS-IV), performance in the training group increased [*χ*^2^(2)=8.769, *p*=0.012] specifically from baseline to post-assessment (*z*=−1.071, *p*=0.005, *r*=0.29). Descriptive statistics hinted toward a slight reduction of performance at follow-up assessment.

### Mindfulness, Affective States, and Questionnaire-Based Cognition

Baseline, post- and follow-up values of questionnaire total scores and analyses of their change over time are shown in Table 7. An overview of subscale scores and change is provided as supplementary data (Appendix Table B).

**Table 7:** Questionnaire total score changes for all measurement points.

Test	Group	Baseline	Post	Follow-Up	Within-group change	
		Median (range)	Median (range)	Median (range)	*χ* ^2^	*p*
AES	TRAINING	31.50 (18–45)	32.00 (18–45)	29.00 (19–42)	2.042	0.360
	WAITLIST	29.50 (19–50)	32.50 (21–46)	33.00 (19–53)	8.982	0.011^*^
BDI-2	TRAINING	10.50 (2–37)	10.00 (0–18)	7.00 (3–19)	5.880	0.053
	WAITLIST	10.50 (1–45)	9.50 (1–48)	10.50 (0–45)	0.107	0.948
CFQ	TRAINING	29.00 (0–78)	24.50 (0–76)	26.00 (1–66)	3.360	0.186
	WAITLIST	30.50 (1–75)	29.00 (3–84)	35.00 (2–81)	7.172	0.028^*^
DEX	TRAINING	16.00 (0–39)	14.50 (0–41)	16.00 (0–43)	0.894	0.640
	WAITLIST	16.00 (3–50)	15.50 (1–50)	18.50 (0–38)	0.441	0.802
FFMQ-D	TRAINING	141.00 (87–162)	150.00 (108–176)	146.50 (116–175)	8.760	0.013^*^
	WAITLIST	139.00 (88–179)	140.50 (109–163)	138.00 (105–173)	0.441	0.802
PDQ-39	TRAINING	23.91 (4–45)	19.97 (0–44)	18.59 (4–46)	4.769	0.092
	WAITLIST	23.05 (6–72)	23.44 (3–80)	28.02 (2–72)	2.459	0.292
PDSS-2	TRAINING	18.00 (6–35)	12.50 (1–34)	16.50 (5–34)	8.000	0.018^*^
	WAITLIST	19.00 (8–37)	20.00 (9–50)	22.50 (9–55)	9.869	0.007^*^
PSQ	TRAINING	27.00 (4–53)	21.00 (4–37)	17.00 (6–37)	4.000	0.135
	WAITLIST	21.00 (0–56)	18.50 (7–55)	19.50 (6–56)	1.782	0.410
QUIP	TRAINING	14.50 (1–22)	10.50 (0–30)	9.50 (0–30)	3.224	0.199
	WAITLIST	9.50 (0–41)	12.50 (0–39)	11.00 (0–39)	1.370	0.504
STAI-S	TRAINING	34.00 (22–63)	32.50 (22–52)	35.00 (26–55)	0.462	0.794
	WAITLIST	36.00 (21–57)	31.00 (27–73)	34.00 (25–60)	0.655	0.712
STAI-T	TRAINING	38.00 (24–71)	37.50 (22–59)	35.50 (24–56)	8.591	0.014^*^
	WAITLIST	35.50 (23–74)	35.00 (25–73)	35.00 (26–75)	0.441	0.802

TRAINING, intervention group; WAITLIST, waitlist-control group; SD, Standard deviation; AES, Apathy Evaluation Scale; BDI-2, Beck Depression Inventory-2; CFQ, Cognitive Failures Questionnaire; DEX, Dysexecutive Questionnaire; FFMQ-D, Five Facet Mindfulness Questionnaire – German Edition; PDQ-39, Parkinson’s Disease Quality of Life Questionnaire-39; PDSS-2, Parkinson’s Disease Sleep Scale-2; PSQ-20, Perceived Stress Questionnaire-20; QUIP, Questionnaire for Impulsive-Compulsive Disorders in PD; STAI-S, State-Trait Anxiety Inventory – subtest state; and STAI-T, State-Trait Anxiety Inventory – subtest trait.

* *p*<0.05.

Apathy scores increased significantly in the control group over time [*χ*^2^(2)=8.982, *p*=0.01]. *Post-hoc* tests revealed significant changes from baseline to post-assessment (*z*=−0.906, *p*=0.01, *r*=0.023) as well as from baseline to follow-up assessment (*z*=−0.781, *p*=0.027, *r*=0.020). Friedman’s test did not show any significant change in the training group.

Overall, questionnaire-based cognitive performance as measured by the Cognitive Failures Questionnaire (CFQ) did not change in the training group but significantly worsened over time in the control group [*χ*^2^(2)=7.172, *p*=0.028]. *Post-hoc* analyses revealed a significant difference of general questionnaire-based cognitive performance from baseline to post-assessment (*z*=−0.875, *p*=0.013, *r*=0.22). Subscale analyses did not reveal any significant change in the control group. Interestingly, subscale forgetfulness changed significantly in the training group [*χ*^2^(2)=7.244, *p*=0.027]. *Post-hoc* tests revealed a significant change from baseline to post-assessment (*z*=0.893, *p*=0.018, *r*=0.24). However, there is a trend of worsening over the course of the follow-up period.

Total mindfulness scores increased in the training group [*χ*^2^(2)=8.760, *p*=0.013] from baseline to post (*z*=−0.857, *p*=0.023, *r*=0.23) and also from baseline to follow-up assessment (*z*=−0.964, *p*=0.011, *r*=0.26). Subscale analyses revealed increases of subscales describing [*χ*^2^(2)=6.565, *p*=0.038], acting with awareness [*χ*^2^(2)=7.172, *p*=0.028] and non-reactivity. For subscale describing, an increase was observed during the training period (*z*=−0.821, *p*=0.03, *r*=0.22) followed by non-significant trend of reduction after the intervention ended. Subscale acting with awareness continuously increased over time [*χ*^2^(2)=7.292, *p*=0.026]. However, significant changes were only found from baseline to follow-up assessment (*z*=−0.893, *p*=0.018, *r*=0.24). Lastly, subscale non-reactivity also increased over the course of the study in the training group [*χ*^2^(2)=7.714, *p*=0.021]. Again, only the increase from baseline to follow-up assessment turned out to be significant (*z*=−0.964, *p*=0.011, *r*=0.26). Neither total mindfulness scores nor subscale scores changed in the control group.

Sleep quality considerably improved over the course of the training in the intervention group [*χ*^2^(2)=8.000, *p*=0.018]. Sleep impairment was reduced from baseline to post-assessment (*z*=0.857, *p*=0.023, *r*=0.023) but returned to its previous state of impairment from post- to follow-up assessment (*z*=−0.857, *p*=0.023, *r*=0.023). In the control group, a continuous trend of worsening sleep quality was observed [*χ*^2^(2)=9.869, *p*=0.007]. Therefore, sleep impairment increased from baseline to post (*z*=−0.906, *p*=0.01, *r*=0.23) and from baseline to follow-up assessment (*z*=−0.969, *p*=0.006, *r*=0.24).

Total scores of the Questionnaire for Impulsive-Compulsive Disorders in PD (QUIP) did not differ within groups at any point in time. Only subscale eating changed within the training group [*χ*^2^(2)=6.500, *p*=0.039]. From baseline to post-assessment, a significant reduction of impulsivity was observed (*z*=0.750, *p*=0.047, *r*=0.20) with a trend of increasing again after the training period ended.

Trait anxiety was significantly reduced in the training group, but not the control group, over the course of the study [*χ*^2^(2)=8.591, *p*=0.014]. Anxiety scores continuously reduced over time, but only the change from baseline to follow-up assessment turned out to be significant (*z*=0.964, *p*=0.011, *r*=0.26). State anxiety did not change within groups at any point.

Neither depression scores, nor perceived stress total or subscale scores did change significantly in any group. Also, there was no significant change of dysexecutive functioning or quality of life total or subscale scores at any point in time.

### Training Feedback and Adherence

Six patients attended all eight training sessions, whereas the overall mean of attended sessions was *M*=7.29 (SD=0.726, Median=7.00). Over the course of the training, patients invested *M*=799.44min (SD=215.72) in formal mindfulness practice at home. Therefore, on average, patients formally practiced mindfulness for about 16min a day. However, only nine of the 14 patients regularly noted their practiced exercises. After training completion, nearly all patients stopped documentation of mindful practices, eliminating adherence statistics from post- to follow-up assessment.

In total, 16 patients returned the feedback questionnaire (two patients were part of the waitlist-control group and participated in the training program after study completion). Twelve patients rated the program as very good and two patients as good (*M*=1.14, SD=0.36, Median=1.00, missing values=2). The audio-CDs were rated as very good by 11 patients and good by three patients (*M*=1.21, SD=0.43, Median=1.00, missing values=2). Most patients found the sessions and practical exercises to be interesting, gained knowledge and were satisfied with the program leader. Nine out of 16 patients rated performing mindfulness exercises at home as well-manageable. However, one patient reported rather poor feasibility and six patients were indecisive. One patient found the training location difficult to reach. A full overview of feedback evaluation is presented in supplementary data (Appendix Table C). Taken together, overall training feedback was very positive.

## Discussion

The main aim of this study was to investigate the effectiveness and feasibility of IPSUM, a newly developed mindfulness program for patients with Parkinson’s disease. Among others, the training program is indeed capable of increasing patients’ mindfulness as well as preventing the worsening of questionnaire-based cognitive impairment and apathy and reducing anxiety levels. Feasibility and patients’ acceptance of the program were very good.

### Primary Outcome

In the present study, about 79% of patients in the training group and a total of 100% of patients in the control group showed signs of ISAm at baseline. Previously, Maier et al. (2016) found signs of ISAm in about 61% of patients in the medication ON-state. In comparison, the frequency of ISAm in the current study of about 90% appears to be higher than previously reported. However, ISA motor impairment values appear to be comparable between both studies (Maier et al.: *M*=8.13, SD=4.30; our sample: *M*=8.63, SD=2.41 in the control group, and *M*=7.75, SD=4.48 in the training group). While Maier et al. (2016) found signs of ISAm-Hypo in 42% of patients, there were about 89% of patients in the control group and about 64% of patients in the training group with ISAm-Hypo in our sample. As percentage scores of ISAm-LID are similar in both studies (Maier et al.: 82%; our sample: 90% in the control group and about 86% in the training group), the big difference of ISAm-Hypo for the control group is quite interesting. It might be possible that the observed frequency of ISAm-Hypo in the control group is an unfortunate result of randomization. Still, considering the whole sample with 21 of 30 patients showing signs of ISAm-Hypo, the percentage of patients with ISAm-Hypo is about 70%. While it is possible that our sample contains a disproportionate amount of patients with ISAm-Hypo, there might be other explanations. It might either indicate that the ISAm assessment tool has difficulties assessing ISAm-Hypo properly or that the underlying mechanisms of ISAm could differ between ISAm-Hypo and ISAm-LID. While it is currently hypothesized that ISAm-LID is caused by impairment of neural processes, ISAm-Hypo might occur because especially mild symptoms are overlooked (Pennington et al., 2020). The current results might partially support this claim due to the big difference of percentages. However, more data are definitely needed to support this claim.

The current study is the first to evaluate ISAm in a longitudinal design. We did not detect any significant change of ISAm or its subscores in any study group over time. As all patients of the control group showed signs of ISAm at baseline and post-assessment and all but one patient at follow-up, we can hardly interpret the results in terms of ISAm changes over time without intervention. Interestingly, statistical analyses did reveal a significant increase of anti-parkinsonism medication and a significant decrease of ISA motor impairment (although no significant change of motor impairment according to the UPDRS was found). It is likely for both changes to be connected to each other and therefore represent a confounding factor. However, despite changes of motor impairment in both groups, total ISAm values did remain quite stable. Even total ISAm percentage scores did not significantly change over time. Because of the low sample size of the current study, we were not able to compute analyses to further examine the impact of LEDD or motor impairment on ISAm. However, future studies should try to maximize their sample sizes in order to evaluate the influence of the aforementioned factors in a longitudinal design.

In conclusion, our results do not support the trainings’ capability of reducing ISAm, which was the primary outcome of this study. There are several possible reasons. Firstly, in regard to the primary outcome, this study might be heavily underpowered, as only 30 patients could be included instead of the approximately 166 patients suggested by a power analysis. Secondly, the applied measurement tool for ISAm has not been tested for its psychometric properties in a longitudinal study design before. As Pennington et al. (2020) concluded, general information about the progression of ISAm in PD is still missing as longitudinal research is unavailable. Also, despite conceptual and potential neurobiological similarities, it remains unclear if ISAm and mindfulness are indeed connected to each other in PD and if this relationship might be influenced by other factors like symptom severity, LEDD, or affective states.

### Secondary Outcomes

Neuropsychological performance mostly did not change within groups, although we were expecting a general improvement of cognitive abilities. However, we found significant improvements in the training group for the WAIS-IV subtest vocabulary from baseline to post-assessment and errors made during the sustained attention task of the TAP which were consistent also at follow-up. We did not find any significant change of cognitive performance for control group patients. Although mindfulness might be a supportive element in the treatment of speech-language pathologies (Medina and Mead, 2020), speech abilities in PD are rarely focused on during examination using a neuropsychological test battery. Cash et al. (2016) previously found an increase in subjectively reported speech abilities after participating in a mindfulness training. Our study partly supports this claim by detecting a mostly objective improvement. Despite the examiner being blinded, the results might still be partly biased as the vocabulary subtest of the WAIS-IV allows for quite a range of subjective interpretation.

In theory, sustained attention is a prerequisite for mindfulness meditation (Lutz et al., 2008). However, previous studies reported conflicted results regarding positive effects of mindfulness training on the ability of sustained attention (Chiesa et al., 2011; Maccoon et al., 2014; Bauer et al., 2020). In our study, the number of errors in the sustained attention task of the TAP was reduced over time in the training group but not the control group. According to the authors, the number of errors can be seen as a secondary criterion in the assessment of sustained attention abilities with number of omissions being the main criterion (Zimmermann and Fimm, 2002). The observed reduction of errors might indicate that patients realized their own unawareness more often over the course of the study. It can therefore be interpreted as an indicator of higher sustained attention, although a definite conclusion on the matter cannot be drawn at this state.

Over time, the training group’s mindfulness scores significantly increased while the control group’s scores remained relatively stable. This applies for general mindfulness level as well as three mindfulness subscores: Describing, Acting with awareness, and Non-reactivity to inner experience. There was no difference for subscales Non-judging of inner experience and Observing. This seems plausible in regard to the subscale Non-judgmental, as patients are constantly asked to evaluate their situation and wellbeing in everyday life, which therefore might be an obstacle. Also, previous studies reported a better fit of a four-factor structure of the Five Facet Mindfulness Questionnaire (FFMQ; instead of a five-factor structure) in clinical and meditation-naïve samples. As this study focused on meditation-naïve patients with PD, the subscale Observing cannot be interpreted accurately (Tables 6 and 7).

Taken together, IPSUM appears to be an effective intervention to increase mindfulness levels in PD patients (FFMQ total score training group increase compared to baseline: +12.3 at post, +13.2 at follow-up). Due to the missing information regarding adherence of mindful practice, it is unclear if patients continued exercising after the last training session. However, this does seem likely due to a slight increase of mindfulness levels between post- and follow-up assessment in the training group.

As expected, groups differed in regard to changes of several negative affective states, including anxiety, apathy, and impulsivity of eating behavior. We assume the reduction of eating impulsivity in the training group may be due to the participants’ positive response to the exercise of mindful eating. Interestingly, apathy scores of the control group worsened from baseline to post-assessment while no significant change was detected in the training group. We assume that mindfulness has been a stabilizing factor preventing an increase of apathy in the training group. Previously, Mele et al. (2021) concluded that mindfulness might indeed be useful to treat symptoms of apathy in PD especially in patients with higher motor impairment and mostly intact cognitive functioning.

In addition, anxiety scores were significantly reduced in the training group, while anxiety levels of the control group remained unchanged. This is in line with research described in the introduction section of this study. Moreover, although change of depression scores failed to reach the level of significance, they also positively changed in the training group on a descriptive level. We hypothesize that a significant effect might have been detected if the sample had not been filtered for depression.

Lastly, a significant finding regarding wellbeing comprised improved sleep quality in the training group from baseline to post-assessment. Although sleep impairment worsened again after the intervention ended, patients of the control group reported a continuous worsening of sleep impairment. This might indicate a relaxing effect of the mindfulness exercises used, as several patients of the training group reported to practice mindfulness in the evening throughout the course of the study.

Another interesting finding is the different progression of self-reported cognitive impairment in daily life (CFQ) in each group. While general cognitive impairment did not significantly change in the training group, patients of the control group reported an increase of impairment at post-assessment compared to baseline assessment. To some extent, mindfulness might have been a stabilizing factor again. A negative correlation between mindfulness and cognitive failures at work has been previously reported for a sample of Australian-based employees (Klockner and Hicks, 2015). Perhaps, this stabilization is partly caused by mindfulness’ positive impact on patients’ anxiety levels. Jankowski and Bąk (2019) identified receptive attention and decentration (two facets of mindfulness) as important mediators of the relationship between anxiety and cognitive failures. Kearney et al. (2016) applied MBSR training to gulf war veterans and found a decrease of cognitive failures after 8weeks. Our results are therefore partially in line with previous studies and support the connection of mindfulness and cognitive failures for the first time in people with Parkinson’s disease.

### Acceptance and Feasibility of the Training Program

To assess patients’ acceptance of a newly conceptualized mindfulness training program, all patients who completed the training protocol were asked to fill out a feedback survey anonymously. Taken together, patients’ feedback was very positive in regard to the training program. All patients rated the sessions as very interesting and mostly well instructed. They felt supported by the program leader and his instructions of the practical exercises. Patients’ satisfaction with the instruction of practical exercises and the training program in general also indicate that the specific adaptations to the specific needs of PD patients were rather meaningful. This supports the need to adapt regular mindfulness programs with certain PD-specific challenges in mind, such as reduced attention span, impaired executive functioning, and lesser mobility.

However, performing the exercises at home might have been difficult for some patients. While the summarizing information material was great, the audio-CDs were partly criticized for not totally supporting feasibility of mindful practice at home. Based on the feedback given, this might be due to issues with the selected titles, which again might have influenced the amount of mindfulness practice at home. For future adaptations of IPSUM, a revision of the audio-CDs’ contents and a more reliable way to track mindfulness practice at home (e.g., *via* smartphone) might therefore be considered.

### Strengths and Weaknesses of This Study

A definitive strength of this this study is the extensive, multimethod test battery, in particular the neuropsychological tests to examine effects of mindfulness training on all five cognitive domains in PD. The newly developed intervention IPSUM proved beneficial in multiple respects and was very well received by the patients. A randomized controlled study design with a blinded rater reduced the chance of bias. Although the number of included patients was significantly smaller than originally planned due to the SARS CoV-2 pandemic, a number of significant changes have been detected. However, due to a missing adherence measure after training completion, the influence of mindful practice after intervention completion is unclear. Also, the follow-up period was only 8weeks which is relatively short. Future studies should include longer follow-up periods to further study long-term effectiveness of IPSUM. The results are limited in terms of generalization and cannot be projected into all patients with PD, as most patients included in this study were classified with Hoehn and Yahr (1967) stage 2. In addition, as we only included patients without severe depression and cognitive impairment to specifically study ISAm, the PD population is only partly represented. Especially patients with depressive symptoms might benefit from taking part in a mindfulness intervention as mindfulness is well known to have a positive impact on mental health. As no active control group was included, the results might partially be influenced by the placebo effect. Due to the limited sample size, no neurobiological data were available to further investigate longitudinal changes. This would have been especially interesting in regard to the main outcome, as the phenomenon of ISAm (and its connection to mindfulness) has not yet been studied using functional MRI data. Unfortunately, we did not collect data in regard to disease duration, which would have been relevant to further examine ISAm-LID as previous studies have found a connection. The smaller sample size also had a negative impact on the general statistical analysis by preventing the use of covariates. Due to the pandemic, the study also suffered an unexpectedly high loss of patients. Therefore, the option of comparing completers and non-completers does not seem reasonable and is not included in the analyses of this study. It also has to be noted that the results might have been different if an intention-to-treat approach had been followed. As we chose to apply the less conservative per-protocol approach to test the efficacy of mindfulness training on ISAm in a controlled sample of PD patients, reported results might be biased.

## Conclusion and Outlook

To the best of our knowledge, this is the first project to study the effects of a mindfulness-based intervention on the phenomenon of ISAm in Parkinson’s disease. While no improvement in regards to self-awareness of motor symptoms in the ON-State was measured, the intervention appears to be capable of increasing the patients’ mindfulness with positive effects in regard to mental health. Additionally, this is the first study to examine the effects of mindfulness training on all five cognitive domains by using an elaborate neuropsychological test battery.

Future studies should include different imaging methods and behavioral assessments in the ON- and OFF-state to better understand the effects of mindfulness training in PD patients and possible connections to ISAm. Moreover, an extensive longitudinal observation of ISAm is needed as an effect of mindfulness training may be detectable with sufficient group sizes. Based on the presented results regarding IPSUM’s effectiveness and feasibility, upcoming research may allow this method to benefit a broader range of PD patients.

## Data Availability Statement

The raw data supporting the conclusions of this article will be made available by the authors, without undue reservation.

## Ethics Statement

The studies involving human participants were reviewed and approved by the University Hospital Marburg (study number: 119/18). The patients/participants provided their written informed consent to participate in this study.

## Author Contributions

TB, CE, and FM contributed to the conception and design of the study. TB developed the training protocol with support of AG, held the training sessions, analyzed the data with support of VJ, FT, and KS, and wrote the first draft of the manuscript. VJ collected the data. All authors contributed to the article and approved the submitted version.

## Funding

The study is funded by the German Parkinson Association.

## Conflict of Interest

In the last 12months, CE has received speaker’s or consulting honoraria from Abbvie Inc., Bial Inc., and Philyra Inc. TB has received consulting honoraria from Philyra Inc. All funders had no role in study design, data collection, decision to publish, or preparation of the manuscript.

The remaining authors declare that the research was conducted in the absence of any commercial or financial relationships that could be construed as a potential conflict of interest.

## Publisher’s Note

All claims expressed in this article are solely those of the authors and do not necessarily represent those of their affiliated organizations, or those of the publisher, the editors and the reviewers. Any product that may be evaluated in this article, or claim that may be made by its manufacturer, is not guaranteed or endorsed by the publisher.
