# Supplementary material for: Pilot Study of Mindfulness Training on the Self-Awareness of Motor Symptoms in Parkinson’s Disease – A Randomized Controlled Trial
Source: Front Psychol. 2021 Nov 30;12:763350. doi: 10.3389/fpsyg.2021.763350 (PMC8670006; doi:10.3389/fpsyg.2021.763350)
Supplement: Supplementary file 1 [file Data_Sheet_1.docx]

| **Outcome** | **Subscale** | **TRAINING** | **WAITLIST** | **Between-group difference** | |
| --- | --- | --- | --- | --- | --- |
|  |  | **Median (range)** | **Median (range)** | **U** | **p** |
| ISAm | Total score | 4.25 (0 – 11) | 5.25 (1 - 11) | 97.50 | .552 |
|  | Dyskinesia | 0.50 (0 – 11) | 1.00 (0 – 5.50) | 102.50 | .697 |
|  | Hypokinesia | 2.50 (0 – 10.50) | 4.25 (0 – 7.50) | 88.50 | .334 |
|  | Tremor right hand | 0.00 (0 – 2.50) | 0.00 (0 - 3) | 115.00 | .918 |
|  | Tremor left hand | 0.00 (0 – .50) | 0.00 (0 – .50) | 121.00 | .728 |
|  | Bradykineisa | 2.00 (0 – 10.50) | 3.75 (0 – 7.50) | 90.00 | .377 |
|  | Motor impairment | 8.50 (1 – 14.50) | 8.25 (5.50 – 14.50) | 101.00 | .667 |
| RWT | Lexical | 18.00 (7 - 27) | 17.00 (4 - 31) | 108.50 | .886 |
|  | Lexical alternating | 21.00 (9 - 36) | 20.50 (14 - 28) | 113.00 | 1.00 |
|  | Semantic | 30.50 (19 - 50) | 30.50 (7 - 50) | 116.00 | .886 |
|  | Semantic alternating | 21.00 (12 - 34) | 19.00 (12 - 27) | 134.50 | .355 |
| TAP | Total omissions | 7.00 (0 - 22) | 4.00 (0 - 21) | 134.50 | .355 |
|  | Total errors | 4.50 (0 - 19) | 2.00 (0 - 28) | 153.50 | .085 |
| VLMT | Learning performance | 39.00 (26 - 61) | 41.00 (29 - 55) | 96.00 | .525 |
|  | Delayed recall | 7.00 (0 - 15) | 8.00 (1 - 12) | 82.50 | .224 |
|  | Recognition | 9.00 (-2 - 15) | 11.00 (-5 - 15) | 85.50 | .275 |
| WAIS | Vocabulary | 45.00 (27 - 57) | 45.50 (14 - 53) | 158.50 | .520 |
| WMS | Spatial forward | 8.00 (4 - 9) | 7.00 (5 - 10) | 138.00 | .294 |
|  | Spacial backward | 6.00 (5 - 9) | 6.50 (4 - 9) | 106.50 | .822 |
| AES | Total score | 31.50 (18 - 45) | 29.50 (19 - 50) | 127.00 | .552 |
| BDI-2 | Total score | 10.50 (2 - 37) | 10.50 (1 - 45) | 106.00 | .822 |
| CFQ | Total score | 29.00 (0 - 78) | 30.50 (1 - 75) | 115.50 | .886 |
|  | Forgetfulness | 13.00 (0 - 31) | 14.00 (1 - 27) | 106.50 | .822 |
|  | Distractibility | 9.50 (0 - 20) | 9.50 (0 - 20) | 105.00 | .790 |
|  | Trigger | 8.00 (0 - 23) | 7.00 (0 - 26) | 130.50 | .448 |
| DEX | Total score | 16.00 (0 - 39) | 16.00 (3 - 50) | 98.00 | .580 |
|  | Inhibition | 4.00 (0 - 13) | 5.50 (2 - 12) | 84.00 | .257 |
|  | Targeted action | 2.50 (0 - 8) | 2.50 (0 - 11) | 94.50 | .473 |
|  | Social regulation | 5.50 (0 - 9) | 6.00 (2 - 14) | 90.00 | .377 |
|  | Abstract thinking | 1.50 (0 - 7) | 2.00 (0 - 12) | 100.50 | .637 |
| FFMQ-D | Total score | 141.00 (87 - 162) | 139.00 (88 - 179) | 97.50 | .552 |
|  | Observing | 26.00 (11 - 31) | 27.50 (16 - 38) | 72.50 | .101 |
|  | Describing | 28.00 (17 - 38) | 27.50 (17 - 40) | 113.00 | 1.00 |
|  | Acting with Awareness | 30.00 (9 - 38) | 29.50 (12 - 37) | 117.50 | .822 |
|  | Non-judging | 32.00 (21 - 38) | 32.00 (12 - 40) | 116.50 | .854 |
|  | Non-reactivity | 21.00 (12 - 29) | 23.00 (18 - 35) | 87.50 | .257 |
| PDQ-39 | Total score | 23.91 (4 - 45) | 23.05 (6 - 72) | 109.00 | .918 |
|  | Mobility | 15.00 (0 - 68) | 30.00 (0 - 83) | 86.00 | .294 |
|  | Activities of daily living | 14.58 (0 - 33) | 14.58 (0 - 92) | 97.50 | .552 |
|  | Emotional well-being | 22.92 (0 - 63) | 22.92 (0 - 88) | 115.50 | .886 |
|  | Stigma | 6.25 (0 - 44) | 3.13 (0 - 25) | 131.00 | .448 |
|  | Social support | 12.50 (0 - 75) | 4.17 (0 - 67) | 125.50 | .580 |
|  | Cognition | 21.88 (0 - 63) | 37.50 (0 - 81) | 76.50 | .142 |
|  | Communication | 16.67 (0 - 67) | 16.67 (0 - 67) | 127.00 | .552 |
|  | Bodily discomfort | 41.67 (0 - 83) | 50.00 (0 - 83) | 102.50 | .697 |
| PDSS-2 | Total score | 18.00 (6 - 35) | 19.00 (8 - 37) | 109.50 | .918 |
| PSQ-20 | Total score | 27.00 (4 - 53) | 21.00 (0 - 56) | 130.50 | .448 |
|  | Worries | 4.50 (0 - 14) | 4.50 (0 - 15) | 121.00 | .728 |
|  | Tension | 7.00 (2 - 15) | 6.00 (0 - 14) | 130.50 | .448 |
|  | Demands | 6.50 (0 - 15) | 5.00 (0 - 12) | 137.50 | .294 |
|  | Joy | 7.00 (3 - 14) | 8.00 (0 - 15) | 95.00 | .498 |
| QUIP | Total score | 14.50 (1 - 22) | 9.50 (0 - 41) | 128.50 | .498 |
|  | Gambling | 0.00 (0 - 0) | 0.00 (0 - 3) | 105.00 | .790 |
|  | Sex | 2.00 (0 - 7) | 0.50 (0 - 8) | 148.50 | .131 |
|  | Buying | 0.50 (0 - 4) | 0.50 (0 - 9) | 114.00 | .951 |
|  | Eating | 1.00 (0 - 9) | 2.00 (0 - 10) | 108.00 | .886 |
|  | Hobby | 3.00 (0 - 7) | 3.00 (0 - 7) | 118.00 | .822 |
|  | Punding | 1.00 (0 - 6) | 0.50 (0 - 6) | 118.50 | .790 |
|  | Medication | 0.00 (0 - 7) | 0.00 (0 - 7) | 114.50 | .918 |
| STAI-S | Total score | 34.00 (22 - 63) | 36.00 (21 - 57) | 112.50 | 1.000 |
| STAI-T | Total score | 38.00 (24 - 71) | 35.50 (23 - 74) | 123.50 | .637 |
| **Appendix Table A: Baseline scores for both groups and baseline difference test results.**  Abbreviations: TRAINING, intervention group; WAITLIST, waitlist-control group; SD, Standard deviation; CFQ, Cognitive  Failures Questionnaire; DEX, Dysexecutive Questionnaire; FFMQ-D, Five Facet Mindfulness Questionnaire – German Ed.;  PDQ-39, Parkinson’s Disease Quality of Life Questionnaire-39; PSQ-20, Perceived Stress Questionnaire-20; QUIP,  Questionnaire for Impulsive-Compulsive Disorders in PD; RWT, Regensburg verbal Fluency test; TAP, test battery for attention;  VLMT, Verbal learning and memory test; WAIS, Wechsler Adult Intelligence Scale – Fourth Edition; WMS, Wechsler Memory  Scale-Revised | | | | | |

| **Outcome** | **Subscale** | **Group** | **Baseline** | **Post** | **Follow-Up** | **Within-group change** | |
| --- | --- | --- | --- | --- | --- | --- | --- |
|  |  |  | **Median (range)** | **Median (range)** | **Median (range)** | **χ²** | **p** |
| CFQ | Forgetfulness | TRAINING | 13.00 (0 - 31) | 9.00 (0 - 31) | 11.50 (0 - 27) | 7.244 | **.027*** |
|  |  | WAITLIST | 14.00 (1 - 27) | 14.00 (3 - 29) | 14.00 (2 - 26) | 1.077 | .584 |
|  | Distractibility | TRAINING | 9.50 (0 - 20) | 7.50 (0 - 19) | 8.50 (1 - 20) | 0.542 | .763 |
|  |  | WAITLIST | 9.50 (0 - 20) | 9.50 (0 - 27) | 10.50 (0 - 25) | 1.849 | .397 |
|  | Trigger | TRAINING | 8.00 (0 - 23) | 6.50 (0 - 23) | 7.00 (0 - 21) | 5.045 | .080 |
|  |  | WAITLIST | 7.00 (0 - 26) | 7.50 (1 - 27) | 8.50 (0 - 28) | 5.922 | .052 |
| DEX | Inhibition | TRAINING | 4.00 (0 - 13) | 4.00 (0 - 13) | 4.00 (0 - 10) | 1.436 | .488 |
|  |  | WAITLIST | 5.50 (2 - 12) | 4.50 (1 - 13) | 5.00 (0 - 10) | 1.265 | .531 |
|  | Targeted action | TRAINING | 2.50 (0 - 8) | 3.00 (0 - 9) | 3.00 (0 - 9) | 0.727 | .695 |
|  |  | WAITLIST | 2.50 (0 - 11) | 3.00 (0 - 10) | 3.00 (0 - 9) | 0.125 | .939 |
|  | Social regulation | TRAINING | 5.50 (0 - 9) | 5.00 (0 - 13) | 5.50 (0 - 14) | 0.780 | .677 |
|  |  | WAITLIST | 6.00 (2 - 14) | 5.00 (1 - 15) | 7.00 (0 - 13) | 0.037 | .982 |
|  | Abstract thinking | TRAINING | 1.50 (0 - 7) | 1.00 (0 - 6) | 1.00 (0 - 9) | 3.692 | .158 |
|  |  | WAITLIST | 2.00 (0 - 12) | 2.00 (0 - 10) | 2.00 (0 - 6) | 2.182 | .336 |
| FFMQ-D | Observing | TRAINING | 26.00 (11 - 31) | 28.00 (13 - 36) | 28.50 (20 - 34) | 5.609 | .061 |
|  |  | WAITLIST | 27.50 (16 - 38) | 28.00 (12 - 36) | 29.50 (11 - 39) | 5.019 | .081 |
|  | Describing | TRAINING | 28.00 (17 - 38) | 33.00 (18 - 38) | 32.00 (16 - 39) | 6.565 | **.038*** |
|  |  | WAITLIST | 27.50 (17 - 40) | 27.00 (15 - 37) | 29.00 (16 - 37) | 1.322 | .516 |
|  | Acting with Awareness | TRAINING | 30.00 (9 - 38) | 32.00 (15 - 39) | 30.50 (18 - 40) | 7.292 | **.026*** |
|  |  | WAITLIST | 29.50 (12 - 37) | 32.00 (17 - 39) | 30.00 (20 - 40) | 3.836 | .147 |
|  | Non-judging | TRAINING | 32.00 (21 - 38) | 33.50 (25 - 40) | 32.00 (28 - 40) | 4.360 | .113 |
|  |  | WAITLIST | 32.00 (12 - 40) | 33.50 (23 - 40) | 32.00 (16 - 39) | 1.433 | .488 |
|  | Non-reactivity | TRAINING | 21.00 (12 - 29) | 23.50 (18 - 33) | 24.50 (13 - 31) | 7.714 | **.021*** |
|  |  | WAITLIST | 23.00 (18 - 35) | 22.50 (13 - 30) | 23.50 (17 - 33) | 4.275 | .118 |
| PDQ-39 | Mobility | TRAINING | 15.00 (0 - 68) | 13.75 (0 - 75) | 11.25 (0 - 53) | 1.105 | .575 |
|  |  | WAITLIST | 30.00 (0 - 83) | 33.75 (0 - 93) | 42.50 (0 - 93) | 3.234 | .198 |
|  | Activities of daily living | TRAINING | 14.58 (0 - 33) | 12.50 (0 - 46) | 12.50 (0 - 54) | 2.098 | .350 |
|  |  | WAITLIST | 14.58 (0 - 92) | 16.67 (0 - 92) | 18.75 (0 - 79) | 5.489 | .064 |
|  | Emotional well-being | TRAINING | 22.92 (0 - 63) | 20.83 (0 - 46) | 20.83 (0 - 46) | 0.178 | .915 |
|  |  | WAITLIST | 22.92 (0 - 88) | 20.83 (0 - 100) | 25.00 (0 - 92) | 2.333 | .311 |
|  | Stigma | TRAINING | 6.25 (0 - 44) | 0.00 (0 - 38) | 0.00 (0 - 50) | 5.448 | .066 |
|  |  | WAITLIST | 3.13 (0 - 25) | 6.25 (0 - 38) | 6.25 (0 - 38) | 3.600 | .165 |
|  | Social support | TRAINING | 12.50 (0 - 75) | 16.67 (0 - 33) | 8.33 (0 - 42) | 0.703 | .704 |
|  |  | WAITLIST | 4.17 (0 - 67) | 0.00 (0 - 75) | 16.67 (0 - 67) | 1.188 | .552 |
|  | Cognition | TRAINING | 21.88 (0 - 63) | 12.50 (0 - 63) | 15.63 (6 - 56) | 3.722 | .155 |
|  |  | WAITLIST | 37.50 (0 - 81) | 37.50 (0 - 88) | 40.63 (0 - 88) | 1.850 | .397 |
|  | Communication | TRAINING | 16.67 (0 - 67) | 12.50 (0 - 58) | 12.50 (0 - 58) | 3.706 | .157 |
|  |  | WAITLIST | 16.67 (0 - 67) | 16.67 (0 - 83) | 16.67 (0 - 58) | 1.190 | .551 |
|  | Bodily discomfort | TRAINING | 41.67 (0 - 83) | 33.33 (0 - 75) | 41.67 (0 - 67) | 2.516 | .284 |
|  |  | WAITLIST | 50.00 (0 - 83) | 41.67 (0 - 100) | 45.83 (0 - 92) | 0.553 | .758 |
| PSQ-20 | Worries | TRAINING | 4.50 (0 - 14) | 4.00 (0 - 8) | 3.50 (0 - 9) | 2.043 | .360 |
|  |  | WAITLIST | 4.50 (0 - 15) | 4.00 (0 - 15) | 4.50 (1 - 14) | 0.160 | .923 |
|  | Tension | TRAINING | 7.00 (2 - 15) | 5.00 (1 - 12) | 5.50 (0 - 13) | 4.512 | .105 |
|  |  | WAITLIST | 6.00 (0 - 14) | 5.00 (0 - 15) | 6.00 (1 - 15) | 2.192 | .334 |
|  | Demands | TRAINING | 6.50 (0 - 15) | 6.50 (0 - 27) | 5.00 (1 - 9) | 1.510 | .470 |
|  |  | WAITLIST | 5.00 (0 - 12) | 5.00 (2 - 12) | 5.00 (1 - 12) | 1.200 | .549 |
|  | Joy | TRAINING | 7.00 (3 - 14) | 7.50 (4 - 40) | 10.00 (5 - 14) | 4.596 | .100 |
|  |  | WAITLIST | 8.00 (0 - 15) | 10.00 (1 - 14) | 10.00 (0 - 15) | 1.192 | .551 |
| QUIP | Gambling | TRAINING | 0.00 (0 - 0) | 0.00 (0 - 2) | 0.00 (0 - 1) | 2.000 | .368 |
|  |  | WAITLIST | 0.00 (0 - 3) | 0.00 (0 - 1) | 0.00 (0 - 3) | 0.000 | 1.000 |
|  | Sex | TRAINING | 2.00 (0 - 7) | 1.50 (0 - 8) | 1.00 (0 - 14) | 1.000 | .607 |
|  |  | WAITLIST | 0.50 (0 - 8) | 0.00 (0 - 10) | 0.00 (0 - 8) | 0.231 | .891 |
|  | Buying | TRAINING | 0.50 (0 - 4) | 1.00 (0 - 4) | 1.00 (0 - 3) | 0.703 | .704 |
|  |  | WAITLIST | 0.50 (0 - 9) | 1.50 (0 - 7) | 2.00 (0 - 7) | 1.024 | .599 |
|  | Eating | TRAINING | 1.00 (0 - 9) | 0.00 (0 - 4) | 1.00 (0 - 6) | 6.500 | **.039*** |
|  |  | WAITLIST | 2.00 (0 - 10) | 3.00 (0 - 11) | 1.50 (0 - 9) | 0.359 | .836 |
|  | Hobby | TRAINING | 3.00 (0 - 7) | 2.50 (0 - 6) | 2.00 (0 - 6) | 0.605 | .739 |
|  |  | WAITLIST | 3.00 (0 - 7) | 2.00 (0 - 11) | 2.50 (0 - 7) | 0.542 | .763 |
|  | Punding | TRAINING | 1.00 (0 - 6) | 2.50 (0 - 6) | 0.50 (0 - 7) | 1.187 | .552 |
|  |  | WAITLIST | 0.50 (0 - 6) | 1.00 (0 - 11) | 0.00 (0 - 5) | 0.619 | .734 |
|  | Medication | TRAINING | 0.00 (0 - 7) | 0.50 (0 - 8) | 1.00 (0 - 5) | 0.974 | .614 |
|  |  | WAITLIST | 0.00 (0 - 7) | 0.00 (0 - 8) | 0.00 (0 - 16) | 1.130 | .568 |
| **Appendix Table B: Questionnaire subscale scores for all measurement points.**  Abbreviations: TRAINING, intervention group; WAITLIST, waitlist-control group; SD, Standard deviation; CFQ, Cognitive Failures Questionnaire; DEX, Dysexecutive Questionnaire; FFMQ-D, Five Facet Mindfulness Questionnaire – German Ed.; PDQ-39, Parkinson’s Disease Quality of Life Questionnaire-39; PSQ-20, Perceived Stress Questionnaire-20; QUIP, Questionnaire for Impulsive-Compulsive Disorders in PD; *, p < 0.05 | | | | | | | |

|  | | | | | |
| --- | --- | --- | --- | --- | --- |
| **Question** | **N** | **M** | **SD** | **Median** | **range** |
| Sessions were interesting | 16 | 2.00 | 0.00 | 2.00 | 2 to 2 |
| Learning growth observed | 16 | 2.00 | 0.00 | 2.00 | 2 to 2 |
| Successful coping with time required | 16 | 1.38 | 0.89 | 2.00 | 0 to 2 |
| Helpful information material | 16 | 1.94 | 0.25 | 2.00 | 1 to 2 |
| Good reachability of training location | 16 | 1.56 | 0.81 | 2.00 | -1 to 2 |
| Great well-being while part of the group | 16 | 2.00 | 0.00 | 2.00 | 2 to 2 |
| Group contributed to personal success | 16 | 1.56 | 0.63 | 2.00 | 0 to 2 |
| Willingness to spend additional time with the group | 16 | 1.56 | 0.63 | 2.00 | 0 to 2 |
| Practical exercises were interesting | 16 | 1.94 | 0.25 | 2.00 | 1 to 2 |
| Practical exercises were well instructed | 16 | 2.00 | 0.00 | 2.00 | 2 to 2 |
| Good feasibility of practical exercises at home | 16 | 0.81 | 0.98 | 1.00 | -1 to 2 |
| Helpful reflection of past session | 16 | 1.69 | 0.70 | 2.00 | 0 to 2 |
| Helpful reflection of past week | 16 | 1.75 | 0.58 | 2.00 | 0 to 2 |
| Moderator’s structure was fine | 16 | 1.94 | 0.25 | 2.00 | 1 to 2 |
| Moderator provided sufficient support | 16 | 2.00 | 0.00 | 2.00 | 2 to 2 |
| Appealing presentation style | 16 | 2.00 | 0.00 | 2.00 | 2 to 2 |
| Felt understood by the moderator | 16 | 2.00 | 0.00 | 2.00 | 2 to 2 |
| Felt good with the moderator | 16 | 2.00 | 0.00 | 2.00 | 2 to 2 |
| Audio-CD supported practical exercise feasibility | 15 | 1.80 | 0.56 | 2.00 | 0 to 2 |
| Audio-CD is important for the training program | 15 | 2.00 | 0.00 | 2.00 | 2 to 2 |
| Titles were selected well | 15 | 1.67 | 0.62 | 2.00 | 0 to 2 |
| Voice of the instructor was enjoyable | 15 | 2.00 | 0.00 | 2.00 | 2 to 2 |
| **Appendix Table C: Feedback evaluation results.**  Abbreviations: M, Mean; SD, Standard deviation | | | | | |
